# Dynamic cell transition and immune response landscapes of axolotl limb regeneration revealed by single-cell analysis

**DOI:** 10.1007/s13238-020-00763-1

**Published:** 2020-08-03

**Authors:** Hanbo Li, Xiaoyu Wei, Li Zhou, Weiqi Zhang, Chen Wang, Yang Guo, Denghui Li, Jianyang Chen, Tianbin Liu, Yingying Zhang, Shuai Ma, Congyan Wang, Fujian Tan, Jiangshan Xu, Yang Liu, Yue Yuan, Liang Chen, Qiaoran Wang, Jing Qu, Yue Shen, Shanshan Liu, Guangyi Fan, Longqi Liu, Xin Liu, Yong Hou, Guang-Hui Liu, Ying Gu, Xun Xu

**Affiliations:** 1 BGI-Shenzhen, Shenzhen, 518083 China; 2 BGI-Qingdao, Qingdao, 266555 China; 3 BGI Education Center, University of Chinese Academy of Sciences, Shenzhen, 518083 China; 4 China National Gene Bank, BGI-Shenzhen, Shenzhen, 518120 China; 5 CAS Key Laboratory of Genomic and Precision Medicine, Beijing Institute of Genomics, China National Center for Bioinformation, Chinese Academy of Sciences, Beijing, 100101 China; 6 University of Chinese Academy of Sciences, Beijing, 100049 China; 7 Institute for Stem Cell and Regeneration, Chinese Academy of Sciences, Beijing, 100101 China; 8 Guangdong Provincial Key Laboratory of Genome Read and Write, BGI-Shenzhen, Shenzhen, 518083 China; 9 National Laboratory of Biomacromolecules, CAS Center for Excellence in Biomacromolecules, Institute of Biophysics, Chinese Academy of Sciences, Beijing, 100101 China; 10 Hubei Key Laboratory of Cell Homeostasis, College of Life Sciences, Wuhan University, Wuhan, 40072 China; 11 State Key Laboratory of Stem Cell and Reproductive Biology, Institute of Zoology, Chinese Academy of Sciences, Beijing, 100101 China; 12 State Key Laboratory of Membrane Biology, Institute of Zoology, Chinese Academy of Sciences, Beijing, 100101 China; 13 Synthetic Biology Technology Innovation Center of Shandong Province, Qingdao, 266003 China

**Dear Editor,**

The axolotl, *Ambystoma mexicanum,* has extraordinary capability to fully recover multiple tissues after lost, whereas such capability has disappeared in mammals. Thus, deciphering detailed mechanisms underlying axolotl regeneration could provide valuable lessons for regenerative medicine. However, many questions, such as the origin of essential progenitor cells and key responses of individual types of cells for regeneration remain elusive (Haas and Whited, 2017). Newly developed single-cell RNA sequencing (scRNA-seq) method enables researchers to observe cellular and molecular dynamics in axolotl regeneration at the single-cell resolution (Gerber et al., 2018; Leigh et al., 2018), but the reported transcriptome landscapes are only for certain cell types or in certain regenerative stages. A complete overview of the regeneration process for all cell types is still lacking.

To this end, we performed axolotl limb amputation followed by scRNA-seq (10× Genomics) on upper forearm tissues at the homeostatic stage (uninjured control, 0 h), and 7 regeneration stages (3 h–33 d) (Fig. 1A, Supplementary Material). In total, we obtained high-quality sequencing data for over 41,000 cells (Table S1). After clustering all cells and reflecting them in uniform manifold approximation and projection (UMAP), we identified 12 putative cell types expressing specific markers (Figs. 1B and S1A; Table S2), which were further confirmed by differentially expressed genes (DEG) analysis (Fig. S1B). By performing integrative comparison of our dataset with ones from Gerber et al. and Leigh et al. (Gerber et al., 2018; Leigh et al., 2018), we found a remarkable similarity of cell-type distribution between our and Leigh et al.’s data (Fig. S2A and S2B), thus further supporting the reproducibility of our experimental procedure.

**Figure 1:**
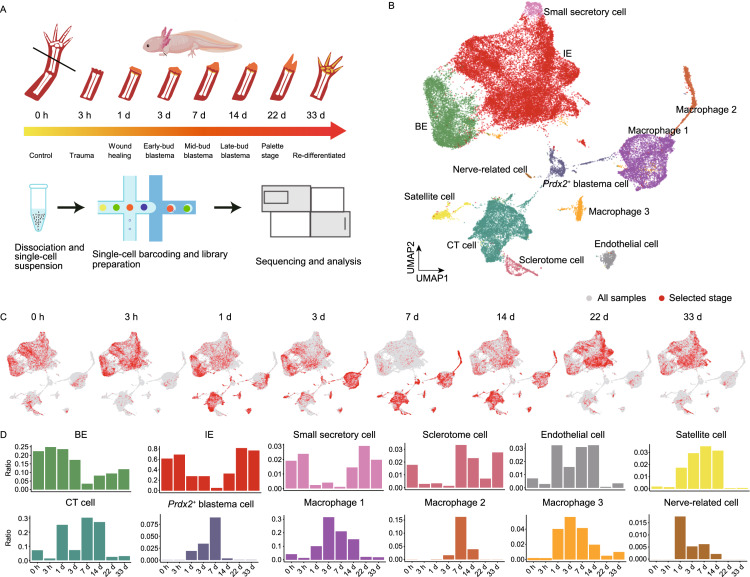

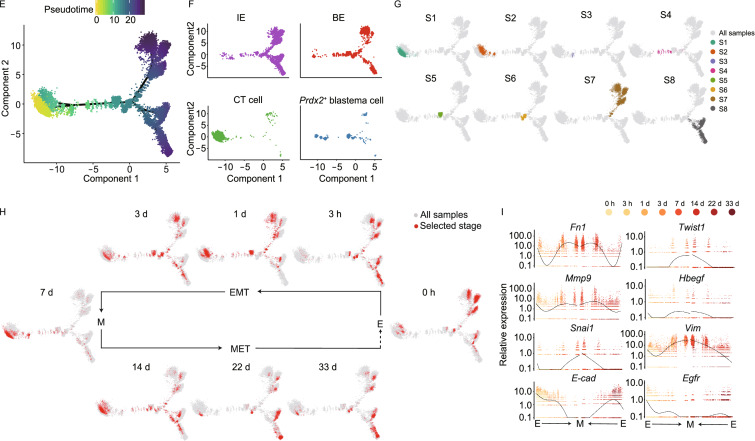
**Integrated transcriptional cell state Atlas of the axolotl limb regeneration revealed a putative EMT-MET process.** (A) Schematic of the scRNA-seq workflow used for the upper arm tissues collected from the homeostatic (uninjured control, 0 h), trauma (3 h), wound healing (1 d), early-bud blastema (3 d), mid-bud blastema (7 d), late-bud blastema (14 d), palette stage (22 d) and re-differentiated stage (33 d). (B) UMAP visualization of the scRNA-seq data from 41,376 single cells collected from 8 sampling stages. Each dot represents a single cell; the colors distinguish clusters. The cell-type annotation is determined by published cell-lineage specific markers. (C) UMAP distribution of cells from each sampling stage. Red dots represent the cells from the corresponding time point, and gray dots represent all the cells from 8 stages. (D) Cell number proportion of each cell type at the corresponding time point. The x-axis represents the time points. The y-axis represents the cell ratio. (E) Pseudotime analysis of epidermal and mesenchymal populations during the regeneration process. Each dot corresponds to a single cell. The gradient bar reflects the pseudotime. The cell-type components are indicated by different colors. (F) Pseudotime analysis reveals the distribution of *Prdx2*^+^ blastema, BE, IE and CT cells along the pseudotime trajectory. The cell-type components are indicated by different colors. (G) *Prdx2*^+^ blastema, BE, IE and CT cells are divided into 8 separate pseudotime states in the pseudotime analysis. Colored dots represent different states. (H) Cell type shift patterns according to real-time points. Red dots represent the cells from the corresponding time point, and gray dots represent all involved cells. (I) Expression of EMT and MET related genes on a single-cell trajectory plot. Colored dots represent single cells from individual sampling time points. Black curves reflect the fitted smooth spline curves

Of note, cells expressing the embryonic limb marker Peroxiredoxin 2 (*Prdx2*) (Gerber et al., 2018) were identified in all three datasets (Fig. S2C). Located at the center of the UMAP plot, *Prdx2*^+^ blastema cells were associated with connective tissue (CT) cells from 1 to 7 d (Fig. 1B and 1C) and expressed genes related to stem cell maintenance, cell morphogenesis and multi-tissue development pathways (Table S3), reinforcing the previous study that this cell type may originate from other types of cells by dedifferentiation, such as CT cells, and may serve as progenitor cells for re-differentiation as previously suggested (Gerber et al., 2018).

More interestingly, the time-course clustering results revealed a clear inverse correlation of cell populations between epithelial, *Prdx2*^+^ blastema and CT cells at two phases (first: 1 d to 7 d; second: 7 d to 22 d) (Fig. 1C and 1D). The number of basal epidermis (BE) and intermediate epidermis (IE) cells, dropped rapidly from 1 d to 7 d and then gradually returned from 7 d to 33 d (Fig. 1C and 1D). In contrast, the number of mesenchymal and *Prdx2*^+^ blastema cells increased in the first phase, and then gradually returned to normal level in the second phase (Fig. 1C and 1D). Such results suggest a potential cell state transformation from epidermal cells into mesenchymal-state cells, and the second phase indicates a reversed process, while it is also possible the rates of cell proliferation/death are distinct between different cell types.

To further evaluate such two possibilities, we pooled different cell populations from various regeneration time points for pseudotime trajectory analysis (Figs. 1E and S3A). While the majority of epidermal (IE and BE) and mesenchymal cell populations were located at two ends of the pseudotime trajectory axis, an evident proportion of both the IE and BE was positioned along the conversion route, and their numbers increased toward the mesenchymal-state end from 1 d to 14 d (Fig. 1F). *Prdx2*^+^ blastema cells were located between the epidermal-state and mesenchymal-state ends with some overlaps (Fig. 1F), suggesting they may possess cellular characteristics of both CT cells and epidermal cells.

To further investigate the cellular correlation, we clustered *Prdx2*^+^ blastema, CT, BE and IE cells into 8 states along the pseudotime axis (Figs. 1G and S3B), and projected them back onto UMAP (Fig. S3C). CT cells were mainly in State 1 (S1) and S2, which are the mesenchymal-like states, while IE and BE mostly located at S7 and S8, which are the epidermal-like states. The intermediate states, S3, S4, S5 and S6, were mixed with CT, epidermal and *Prdx2*^+^ blastema cells (Figs. 1G and S3B). At individual sampling time point, the cell number in the epidermal-like states continuously dropped from 1 d to 7 d, while the cell number in the intermediate and mesenchymal-like states gradually increased (Fig. 1G and 1H) and reached their peaks at 7 d (Fig. 1C, 1D and 1H). From 14 d to 33 d, as the number of mesenchymal cells decreased, epidermal cells were repopulated (Fig. 1C, 1D and 1H). Interestingly, a significant portion of IE and BE with the epidermal-like state transformed into the intermediate-like state from 1 d to 7 d (Fig. S3B), and while most of those cells remained in the intermediate state, a minor part further transformed into mesenchymal-like state at 7 d and 14 d (Fig. S3B). At last, those cells shifted back into an epidermal-like state from 14 d to 33 d (Fig. S3B). Altogether, these digital cell state transitions provide an extra possibility for the changes in cell number of epidermal and mesenchymal tissues, in addition to variant cell proliferation/death rate, suggesting that there is a sequential epithelial to mesenchymal transition (EMT), followed by a mesenchymal to epithelial transition (MET) during limb regeneration.

Therefore, key EMT genes (Kalluri and Weinberg, 2009; Zhang et al., 2013) were then analyzed to further confirm this process. The expression patterns of EMT genes showed a similar tendency with the cell population distribution, as the expression levels of genes positively related to EMT continued to increase after limb amputation and peaked at 7 d, and then reduced after 14 d (Fig. 1I). In contrast, genes negatively related to EMT, including cadherin 1 (*E-cad*) and epidermal growth factor receptor (*Egfr*), exhibited the exact opposite pattern (Fig. 1I). These facts, complied with our cellular dynamics data, provide evidence of a potential dynamic EMT-MET process in axolotl limb regeneration with turning point between 7 d and 14 d. However, since epithelial and CT cells are both linked to the *Prdx2*^+^ blastema cells, it is difficult to distinguish between two possibilities that epidermal cells directly transformed into CT lineage cells, and that the *Prdx2*^+^ blastema cells functioned as a transient stage linking these two groups of cells.

It has been established that EMT plays a critical role in physiologic tissue repair, yet sustained EMT promotes fibrosis of multiple organs under a variety of pathological conditions (Stone et al., 2016; Forte et al., 2017). By examining the co-expression of marker genes, our data revealed that cells characterized as fibroblasts were present during axolotl limb regeneration, most of which were in CT cluster (Fig. 2A). Importantly, the number of this group of cells also first increased, peaked at 7 d and 14 d, and then decreased till absence. In contrast, during digit wound healing process of mouse, the number of fibroblasts at the wound area appeared to be consistent and relatively high at all stages according to our scRNA-seq data (Figs. 2A, S4A and S4B). Altogether, axolotl limb regeneration seems to experience a temporal EMT, which may prevent uncontrolled fibrosis.

**Figure 2:**
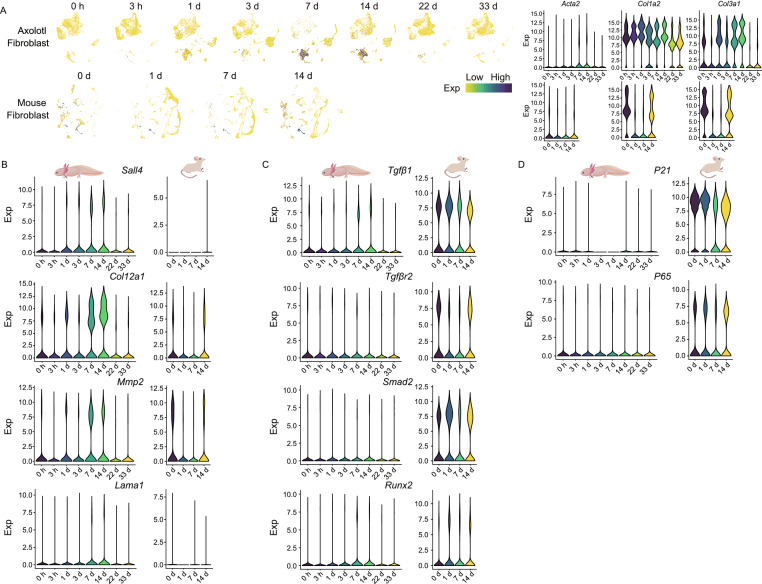

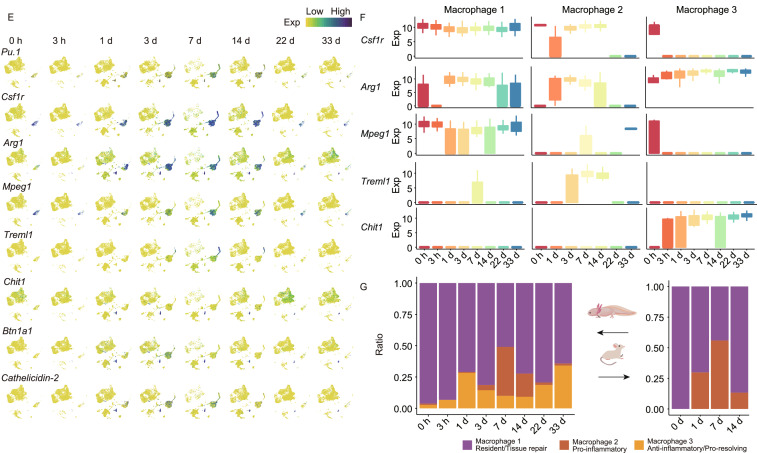
**The cellular and molecular dynamics of ECM and immune microenvironment during axolotl limb regeneration.** (A) UMAPs reflect the cellular distribution of fibroblasts during different stages of axolotl limb regeneration and mouse digit wound healing. Each dot represents a cell. Violin plots reflect the expression of the cell type identify markers. (B) Violin plots reflect the gene expression of ECM deposition and modeling related genes during axolotl limb regeneration and mouse digit wound healing. (C) Violin plots reflect the gene expression of fibrosis pathway related genes during axolotl limb regeneration and mouse digit wound healing. (D) Violin plots reflect the gene expression of cellular senescence related genes during axolotl limb regeneration and mouse digit wound healing. (E) Expression and distribution of marker genes and key genes of macrophages. Each dot represents a cell, and the color corresponds to the expression level (log_2_(CPM + 1)). (F) Expression of macrophage marker genes in each macrophage cluster at different time points. Error bars indicate the standard deviation to the expression level (log_2_(CPM + 1)). (G) Cell number distribution of the macrophage subtypes at each time point during axolotl limb regeneration (left) and mouse digit wound healing (right)

Consistently at the molecular level, the expression levels of extracellular matrix (ECM) deposition related genes, including spalt like transcription factor 4 (*Sall4*) (Erickson et al., 2016) and collagen type XII alpha 1 chain (*Col12a1*) and ECM remodel related genes, including matrix metallopeptidase 2 (*Mmp2*) and laminin subunit alpha 1 (*Lama1*) (Rayagiri et al., 2018) were low at the beginning of axolotl regeneration, but increased and peaked at 7 d and 14 d (Fig. 2B). In contrast, the same genes in the healing mouse digits were low or undetectable throughout the healing process (Fig. 2B). This result suggests that different from mouse wound healing, axolotl simultaneously undergoes a rapid ECM redeposition and remodeling, leading to a complete ECM recovery.

To further explore the mechanism of gene regulation for fibrosis, we examined the expression of key genes in fibrogenic transforming growth factor beta (TGF-β) pathway (Meng et al., 2016). We observed the expression level of *Tgf-β1* was relatively low and only increased at 7d and 14d during axolotl regeneration, while the expression of TGF-β1 receptor, transforming growth factor beta receptor 2 (*Tgfβr2*) and downstream factors RUNX family transcription factor 2 (*Runx2*) and SMAD family member 2 (*Smad2*) remained consistently low at all stages (Fig. 2C). In sharp contrast, in the mouse model, cells that expressed these genes were greater in number and showed a wider distribution over time than those in axolotl (Fig. 2C). Also, as another downstream consequence of TGF-β pathway, the expression levels of cell senescence markers cyclin dependent kinase inhibitor 1A (*P21*) and nuclear factor of kappa light polypeptide gene enhancer in B-cells 3 (*P65*) were constantly low, whereas in mouse cells the expression of these genes remained high throughout the wound healing process. Altogether, our results strongly suggest that fibrosis and cellular senescence are restrained at minimal level in regenerating axolotl cells. The low level of TGF-β pathway activity, together with unique ECM redeposition and remodel pattern, may contribute to the scarless regeneration of axolotl limbs.

Macrophages play critical roles during fibrosis and scar formation, and thus are key players in regulating wound healing and tissue repair (Stone et al., 2016). Godwin et al. reported that macrophages are required for proper limb regeneration in axolotl and showed that systemic macrophage depletion resulted in scarred wound closure and the permanent failure of limb regeneration (Godwin et al., 2013). In our data, three clusters of macrophages were distinguished by the differential expression of marker genes during regeneration (Figs. 1B, 2E and 2F). The expression pattern of marker genes of Macrophage 1 was similar to that in the reported macrophage types with known tissue repair and anti-bacterial functions (Fig. 2E and 2F; Table S2) (Wynn and Vannella, 2016). The distribution of Macrophage 1 was also found overlapped with Macrophage and Recruited Macrophage identified by Leigh et al. (2018) in our integrated analysis (Fig. S5A and S5B). Macrophage 2 expressed markers similar to the typical pro-inflammatory macrophage (Fig. 2E and 2F; Table S2) (Wynn and Vannella, 2016). Macrophage 3 appeared to be a type of pro-resolving and anti-inflammatory macrophage according to its marker gene expression pattern (Fig. 2E and 2F; Table S2), and its expression of both anti-infection and anti-inflammatory genes (Table S4). In addition, the majority of our Macrophage 3 cells were found overlapped with neutrophils identified by Leigh et al. (2018) (Fig. S5C), possibly due to their shared expression patterns in genes related to anti-inflammation function (Marwick et al., 2018). To our interest, DEG analysis showed that the expression of Nuclear Factor Kappa B (*Nf-κb*) was decreased in Macrophage 3 from 1 d to 7 d (Table S4). Once activated, NF-κB acts as a key transcription factor that induces expression of inflammatory mediators to enrich the pro-inflammatory macrophage phenotype (Doyle and O'Neill, 2006; Sharif et al., 2007). Inactivation of NF-κB suppresses the release of inflammatory cytokines, thus generating anti-inflammatory/pro-resolving macrophage phenotype (Marwick et al., 2018).

Macrophages are required for wound healing or epimorphic regeneration in mammals, in which inflammatory macrophages play an important role in stimuli response in the early stages before weakened and replaced by pro-resolving macrophages at the wound site (Wynn and Vannella, 2016). Such a time-dependent process greatly promotes fibrosis and scarring at the wounding site, ensuring wound closure and healing in mouse. Our mouse digit wound healing model confirmed that only Macrophage 1 and Macrophage 2 were observed at the early stages in injured mouse digit (Figs. 2G, S4A and S4B), implying a need for inflammatory and immune wound healing responses. In contrast, axolotl limb regeneration seemed to utilize an additional mechanism to balance the inflammatory response by recruiting a third type of pro-resolving-like macrophage to the site of limb structure recovery at early stages (Fig. 2G). Only a few Macrophage 2 at the wound site were observed in the initial and early stages of axolotl regeneration until 7 d (Figs. 1D and 2G). Instead, Macrophage 1 and Macrophage 3 appeared to respond to injury quickly and accumulated as early as 1 d, until their cell number decreased after 14 d (Figs. 1D and 2G). Both Macrophage 2 and Macrophage 3 expressed high levels of cell migration factors as well as proliferation-regulating factors (Fig. S6A and S6B), implying that accumulation of these macrophages at regeneration site might be due to both recruitment and proliferation. Whether the accumulation of Macrophage 3 would be at least partially responsible for the suppression of the inflammation and fibrosis that leads to scar formation, warrants further functional investigation.

In sum, our scRNA-seq covers the regenerative process from the immediate response stage until the complete recovery stage, providing data for a thorough landscape of a highly dynamic cell reprogramming and microenvironment. In comparison to the mouse wound healing process, we report here a unique molecular feature in axolotl that may contribute to the complete regeneration, containing a sequential EMT and MET process followed by a unique temporal ECM reconstruction and suppressed activation of fibrosis and cellular senescence pathways. This cell fate transition process is also linked to unique immune responses mediated by three types of macrophages, mechanistically different from that in mouse. Our data provide valuable mechanistic and biological insights into how multi-tissue structures regenerate in a spatial–temporal manner, which may guide future efforts in regenerative medicine.

## Electronic supplementary material

Below is the link to the electronic supplementary material.

Supplementary file1 (PDF 17303 kb)

Supplementary file2 (XLSX 98 kb)
