# Supplementary material for: Dynamic cell transition and immune response landscapes of axolotl limb regeneration revealed by single-cell analysis
Source: Protein Cell. 2020 Aug 3;12(1):57–66. doi: 10.1007/s13238-020-00763-1 (PMC7815851; doi:10.1007/s13238-020-00763-1)
Supplement: Supplementary file 1 — Supplementary file1 (PDF 17303 kb) [file 13238_2020_763_MOESM1_ESM.pdf]

Supplementary Materials

Materials and Methods

Animal husbandry and operation

The axolotl (*Ambystoma mexicanum*) cultivation and experimental methods were performed in accordance with the guidelines of the ethics committee of BGI (permit BGI-IRB 19059). In this study, we used male and female D/D strain axolotls with a length between 8 and 10 cm from snout to tail. For amputation surgery, axolotls were anesthetized with 0.2% Tricaine (ethyl 3-aminobenzoate methane sulfonate). Mice were ordered from Charles River and housed at the Institute of Biophysics, Chinese Academy of Sciences, in a SPF facility. The laboratory was under controlled temperature (20-25°C), humidity (30%-70%) and light (12-hour light-dark cycle). All animal experiments were conducted in compliance with animal protocols approved by the Chinese Academy of Sciences Institutional Animal Care and Use Committee.

Amputation and dissociation of axolotl upper arms for scRNA-seq

Five forearm tissues from different axolotls were pooled together for library construction, for the homeostatic (uninjured control, 0 h), and 7 regeneration time points, including trauma (3 hours post amputation, 3 h), wound healing (1 day post amputation, 1 d), early-bud blastema (3 d), mid-bud blastema (7 d), late-bud blastema (14 d), palette (22 d) and re-differentiation (33 d) stages. Homeostatic sample was collected from amputated upper forearm, while other tissue samples were collected from the amputation wound. For stages before 3d, incision was made at 0.5 mm proximally of the wound site. For stages after 7d, incision was made at the wound site to collect the entire blastema. Each sample was physically separated by tissue type, and then minced and digested in a 0.2% mixture of collagenase I, collagenase II (BBI) and collagenase IV, diluted in 0.8× DPBS at 25°C for 1 hour by being shaken and pipetted gently every 15 minutes. Dispase II and pancreatin were then added to the mixture at a final concentration of 1 U mL^{-1}, and the sample was digested at 25°C again. Cells were harvested and suspended after filtration with a 40 µm cell strainer. Cell pellets were centrifuged at 1,500 rpm for 5 minutes before collection. The screening, washing and centrifugation steps were repeated multiple times before the cell pellets were finally resuspended in 1× PBS (calcium and magnesium free) containing 0.04% BSA (400 µg mL^{-1}) to generate the single cell suspension used for the library construction.

Amputation and dissociation of mouse upper arms for scRNA-seq

Five tissues from different mice were pooled together for library construction for each regeneration time point. Each sample was minced and digested in a 0.2% mixture of collagenase I and collagenase IV diluted in 1× DPBS at 37°C for 1 hour while being shaken and pipetted gently every 15 minutes. Dispase II and pancreatin were then added to the mixture at a final concentration of 1 U mL^{-1}, and the sample was digested at 37°C again. Thirty minutes later, the digestion was stopped by 10% FBS in PBS. The mixture was filtered by a 40 µm cell strainer before centrifugation at 400 g for 6 minutes. Cells were suspended in red blood cell lysis buffer for 5 minutes and centrifuged at 400 g for 6 minutes again before collection. The cell pellets were screened by flow cytometry and finally collected in 1× PBS (calcium and magnesium free) containing 0.04% BSA (400 µg mL^{-1}) to generate the single cell suspension used for the library construction.

Single-cell RNA-seq (scRNA-seq)

The scRNA-seq library was constructed using the Chromium single-cell 3 prime v2 reagent kit (10× Genomics) according to the manufacturer's instructions (https://support.10xgenomics.com/single-cell-gene-expression/index/doc/user-guide-chromium-single-cell-3-reagent-kits-user-guide-v2-chemistry). All libraries were further prepared based on the requirements of the BGISEQ-500 sequencing platform manufactured by MGI®. The DNA concentration was determined by a Qubit (Invitrogen®). Then, samples with 2 pmol of nucleotides were pooled to generate single-strand DNA circles (ssDNA circles). DNA nanoballs (DNBs) were generated with the ssDNA circles by rolling the circles during replication to significantly increase the fluorescent signals during the sequencing process. The DNBs were loaded into the patterned nanoarrays and sequenced on the BGISEQ-500 sequencing platform with a paired end read length of 26-100 bp.

Gene reannotation

To obtain the comprehensive genetic annotation information, we reannotated the transcriptome sequence using the protein sequence from the NCBI and UniProt. The human (GRCh38), mouse (GRCm38), *gallus gallus domesticus* (GRCg6a), zebrafish (GRCz11) and *xenopus laevis* (V2) protein sequences were downloaded from the NCBI, while the SwissProt and human marker databases were downloaded from UniProt. We then aligned all the public data against the axolotl transcriptome by using blastall(Altschul et al., 1990) (version 2.2.25) with the main parameter of "–e 1e-5". The best hit result from alignments with a Z-score≧ 200 was used to perform the homologous annotation.

scRNA-seq data processing

The average number of reads per sample of scRNA-seq data was 667,250,032, and median number of genes per cell was 1,433 (Table S1). The data were aligned to the axolotl genome(Nowoshilow et al., 2018) (https://www.axolotl-omics.org/assemblies) with STAR(Dobin et al., 2013), which was implemented in the 10× Genomics Cell Ranger-2.0.1 software with default parameters to generate the absolute Unique Molecular Identifier (UMI) counts. For the alignment, the ribosomal protein genes were excluded from all datasets. The resulting UMI counts generated the gene-cell matrices used for the downstream analysis; the matrices initially contained 23,106 unique genes from all samples and a mean of ~ 103,260 reads per cell in each sample.

Unbiased clustering with Seurat

The cell-gene matrices were filtered to retain genes expressed in at least five cells from each sample. Cells with low gene expression (< 500) were also removed. Filtered data were imported into R and integrated by using Seurat(Stuart et al., 2019) (version 3.0.3). Downstream analyses, such as normalization, shared nearest neighbor graph-based clustering, and differential expression analysis and visualization, were performed by using the R package Seurat (version 3.0.3). To enhance the identification of common cell types and enable comparative analyses of different stages, we integrated datasets from all stages by using the 'FindIntegrationAnchors' function in Seurat, which was implemented in the Seurat workflow (https://satijalab.org/seurat/v3.0/immune_alignment.html). The clusters were identified using a community identification algorithm that was implemented in the Seurat 'FindClusters' function. The resolution parameter used to find the resulting

number of clusters was set to 0.3 to produce an appropriate number of clusters that were large enough to capture most of the biological variability. The clustering results were presented using a uniform manifold approximation and projection (UMAP) based on plots generated by the 'RunUMAP' function. The 'FindAllMarkers' function was used to identify differentially expressed genes with default parameters.

DEG analysis of the regeneration stages

Each regenerative stage was compared with the control to evaluate the gene expression changes over time, and the differentially expressed genes (DEGs) were identified by the 'FindMarkers' function in Seurat (version 3.0.3). The DEGs were filtered by using |log2(foldchange)| > 1 and adjusted with a P value < 0.01. The combined mean expression of the DEGs in each cell type at different stages was calculated, and the results were shown in Figure S1B.

DEGs were divided into 12 clusters according to the parameter cutree_rows = 12 based on hierarchical clustering in the pheatmap (version 1.0.10) package (https://cran.r-project.org/web/packages/pheatmap/index.html). The GO terms associated with each cluster were analyzed by the 'enrichGO' function (parameters OrgDb = org.Hs.eg.db, ont = "BP", pAdjustMethod = "BH") in the clusterProfiler (version 3.10.0) package(Yu et al., 2012).

Single-cell trajectory construction

For EMT (epithelial to mesenchymal transition) and MET (mesenchymalto epithelial transition) related cells (intermediate epidermis, basal epidermis, connective tissue, blastema, satellite cells, and sclerotome cells), we used Monocle(Trapnell et al., 2014) (version 2.10.0) to construct the single-cell trajectory for each defined cell type with known markers. Highly variable genes with q value < 0.01 were selected, and a discriminative dimensionality reduction (DDRTree) was performed with regression according to the UMI counts to eliminate unwanted variation introduced by variation in the sequencing depth between samples, and the cell states were ordered along the trajectory according to their pseudotime values.

Supplementary References

Altschul, S.F., Gish, W., Miller, W., Myers, E.W., and Lipman, D.J. (1990). Basic local alignment search tool. Journal of molecular biology 215, 403-410.

Dobin, A., Davis, C.A., Schlesinger, F., Drenkow, J., Zaleski, C., Jha, S., Batut, P., Chaisson, M., and Gingeras, T.R. (2013). STAR: ultrafast universal RNA-seq aligner. Bioinformatics 29, 15-21.

Nowoshilow, S., Schloissnig, S., Fei, J.F., Dahl, A., Pang, A.W.C., Pippel, M., Winkler, S., Hastie, A.R., Young, G., Roscito, J.G., *et al.* (2018). The axolotl genome and the evolution of key tissue formation regulators. Nature 554, 50-55.

Stuart, T., Butler, A., Hoffman, P., Hafemeister, C., Papalexi, E., Mauck, W.M., 3rd, Hao, Y., Stoeckius, M., Smibert, P., and Satija, R. (2019). Comprehensive Integration of Single-Cell Data. Cell 177, 1888-1902.e1821.

Trapnell, C., Cacchiarelli, D., Grimsby, J., Pokharel, P., Li, S., Morse, M., Lennon, N.J., Livak, K.J., Mikkelsen, T.S., and Rinn, J.L. (2014). The dynamics and regulators of cell fate decisions are revealed by pseudotemporal ordering of single cells. Nature biotechnology 32, 381-386.

Yu, G., Wang, L.G., Han, Y., and He, Q.Y. (2012). clusterProfiler: an R package for comparing biological themes among gene clusters. Omics : a journal of integrative biology 16, 284-287.

Figure S1. Identification of Cell Types Corresponding to the 12 scRNA-seq Clusters Based on Established Markers of Cell Identity. (A) Barplots reflect expression of cell-type marker genes used for cell lineage identification. X-axis: cell clusters. Y-axis: expression represented by $\log_2$(CPM+1). Error bars indicate the standard deviation. (B) Heatmap reveals the scaled mean expression of DEGs at different time points, with genes indicated by rows. Color bars on the X-axis represents the cell types. The color bar on the Y-axis represents the DEG modules, and each colored module is a set of genes with similar DEG pattern.

Figure S2. Integrated Analysis with Datasets from this Study, Gerber et al. and Leigh et al., for *Prdx2*$^+$ Cells. (A) Cell distribution in UMAP of three datasets after integration. (B) Cell distribution in UMAP at different stages of three datasets after integration. (C) Distribution in UMAP of *Prdx2*$^+$ cells in three datasets. Depth of color demonstrates expression level, as exhibited in scale bar. BGI means data generated from this study, WL means data from Leigh et al., TL means data from Gerber et al.

Figure S3. Dynamics of EMT and MET Related Cell Populations during the Regeneration Process. (A) Pseudotime analysis revealed the distribution of *Prdx2*$^+$ blastema, BE, IE, CT, satellite and sclerotome cells along the pseudotime trajectory. (B) Substrate distribution in the IE, BE, and CT and *Prdx2*$^+$ blastema cells at 8 sampling time points. Cells in different states in the pseudotime trajectory map are represented by different colors. (C) Projection of 8 pseudotime states onto UMAP. Colors represent different pseudotime states.

Figure S4. Cell Clustering and Cell Type Identification Markers in Wound Healing Mice. (A) UMAP visualization of cells collected at homeostasis, 1 d, 7 d and 14 d from a mouse digit wound healing model. (B) Expression of cell marker genes in mice was used to identify cell types. Each dot represents a cell, and the color corresponds to the expression level ($\log_2$(CPM+1)).

Figure S5. Integrated Analysis with Datasets from this Study, Gerber et al. and Leigh et al., for Immune Cells. (A) Merged cell-type distribution in UMAP of immune cells of three datasets after integration. (B) Isolated cell type distribution in UMAP of immune cells of three datasets. (C) Comparison of expression pattern of Macrophage 3 marker genes, between datasets of BGI and Leigh et al. Depth of color demonstrates expression level, as exhibited in scale bar. BGI represents data from us, WL represents data from Leigh et al., TL represents data from Gerber et al.

Figure S6. The Molecular Features of Macrophages. (A) Heatmap visualization of regeneration-related pathways with genes in rows and cells in columns. Color bars on the X-axis: the first row represents the sampling time points, and the second row represents the cell types. The color bar on the Y-axis represents the pathways. (B) Expression and distribution of genes related to migration (Actin Related Protein 3 (*Actr3*), Ras Homolog Family Member A (*Rhoa*)), tyrosine kinase adaptor (LYN Proto-Oncogene Src Family Tyrosine Kinase (*Lyn*)) and proliferation (Glia Maturation Factor Gamma (*Gmfg*)) pathways. Each dot represents a cell, and the color corresponds to the expression level ($\log_2$(CPM+1)).

Figure S1

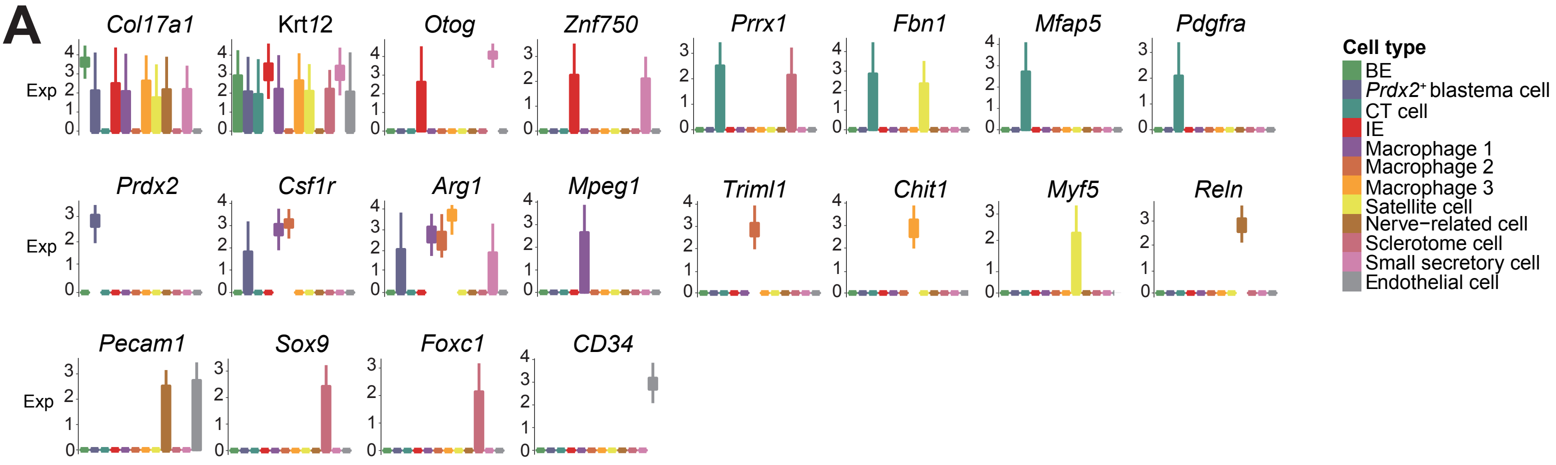
A
Col17a1
Krt12
Otog
Znf750
Prrx1
Fbn1
Mfap5
Pdgfra
Prdx2
Csf1r
Arg1
Mpeg1
Triml1
Chit1
Myf5
Reln
Pecam1
Sox9
Foxc1
CD34
Exp
Cell type
BE
Prdx2+ blastema cell
CT cell
IE
Macrophage 1
Macrophage 2
Macrophage 3
Satellite cell
Nerve−related cell
Sclerotome cell
Small secretory cell
Endothelial cell

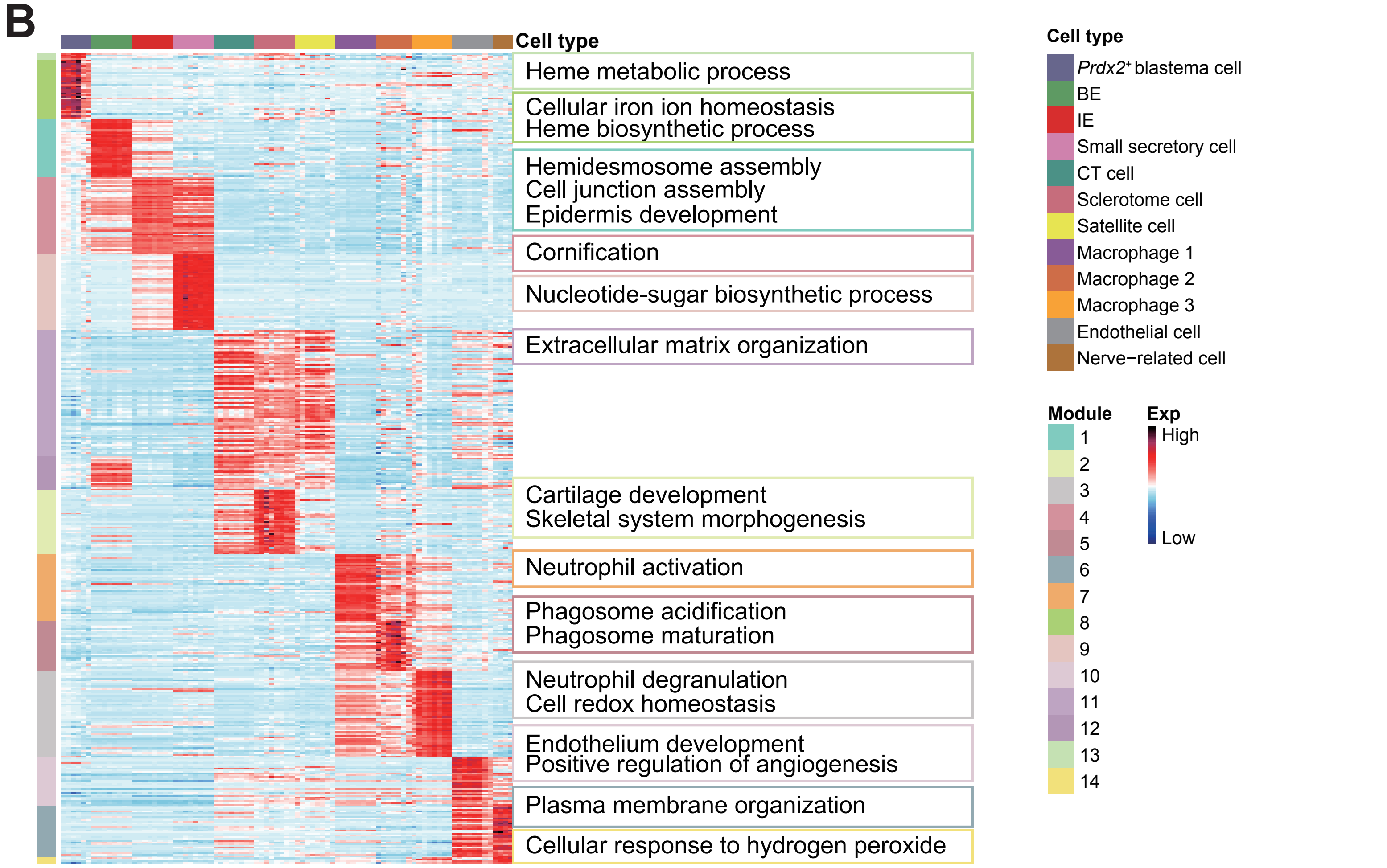
B
Cell type
Heme metabolic process
Cellular iron ion homeostasis
Heme biosynthetic process
Hemidesmosome assembly
Cell junction assembly
Epidermis development
Cornification
Nucleotide-sugar biosynthetic process
Extracellular matrix organization
Cartilage development
Skeletal system morphogenesis
Neutrophil activation
Phagosome acidification
Phagosome maturation
Neutrophil degranulation
Cell redox homeostasis
Endothelium development
Positive regulation of angiogenesis
Plasma membrane organization
Cellular response to hydrogen peroxide
Cell type
Prdx2+ blastema cell
BE
IE
Small secretory cell
CT cell
Sclerotome cell
Satellite cell
Macrophage 1
Macrophage 2
Macrophage 3
Endothelial cell
Nerve−related cell
Module
1
2
3
4
5
6
7
8
9
10
11
12
13
14
Exp
High
Low

Figure S2

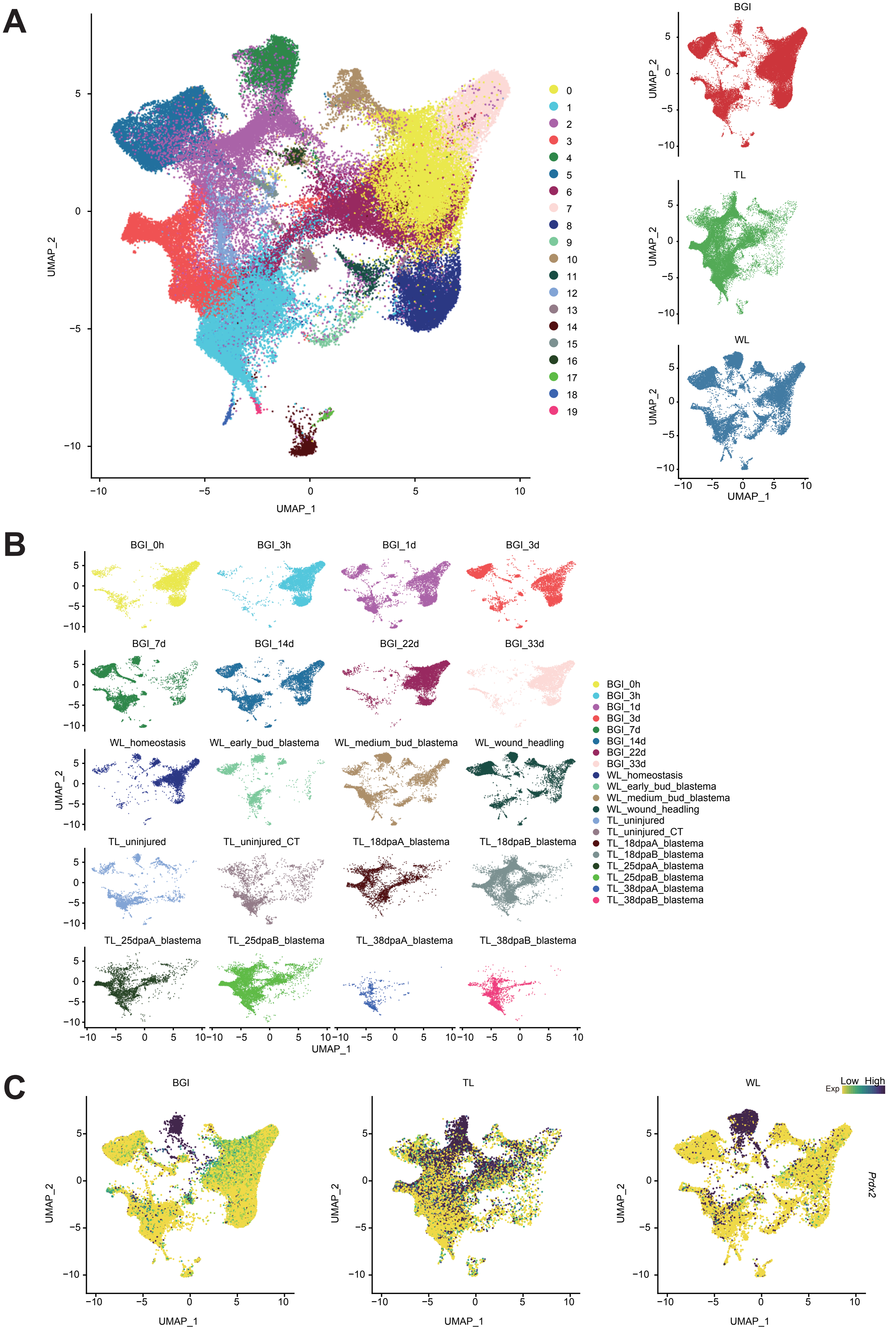
A
UMAP_1
UMAP_2
0
1
2
3
4
5
6
7
8
9
10
11
12
13
14
15
16
17
18
19
BGI
TL
WL
B
BGI_0h
BGI_3h
BGI_1d
BGI_3d
BGI_7d
BGI_14d
BGI_22d
BGI_33d
WL_homeostasis
WL_early_bud_blastema
WL_medium_bud_blastema
WL_wound_headling
TL_uninjured
TL_uninjured_CT
TL_18dpaA_blastema
TL_18dpaB_blastema
TL_25dpaA_blastema
TL_25dpaB_blastema
TL_38dpaA_blastema
TL_38dpaB_blastema
C
Exp
Low
High
Prdx2

Figure S3

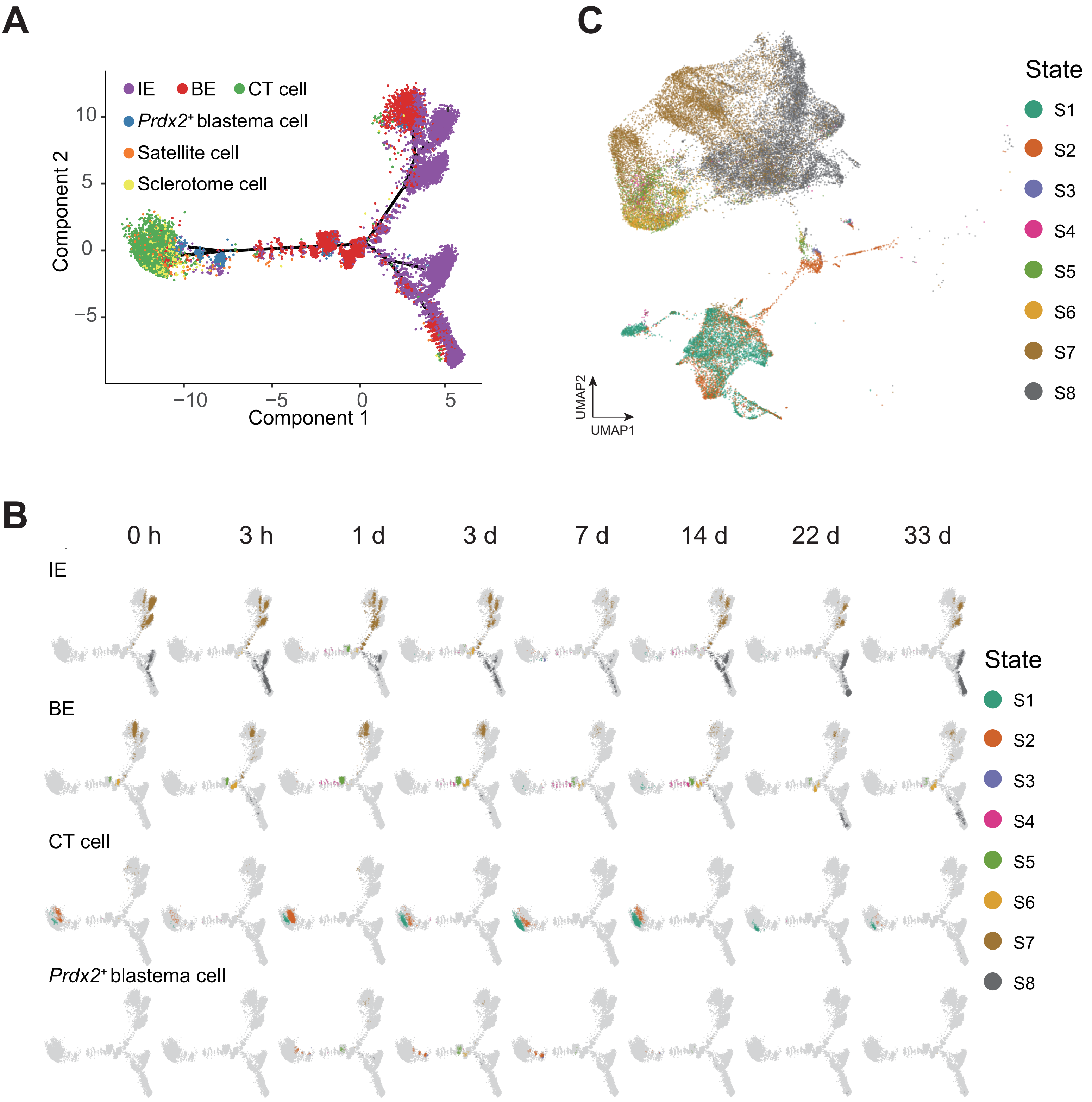
A
C
IE
BE
CT cell
Prdx2⁺ blastema cell
Satellite cell
Sclerotome cell
Component 2
Component 1
10
5
0
−5
−10
State
S1
S2
S3
S4
S5
S6
S7
S8
UMAP2
UMAP1
B
0 h
3 h
1 d
3 d
7 d
14 d
22 d
33 d
IE
BE
CT cell
Prdx2⁺ blastema cell
State
S1
S2
S3
S4
S5
S6
S7
S8

Figure S4

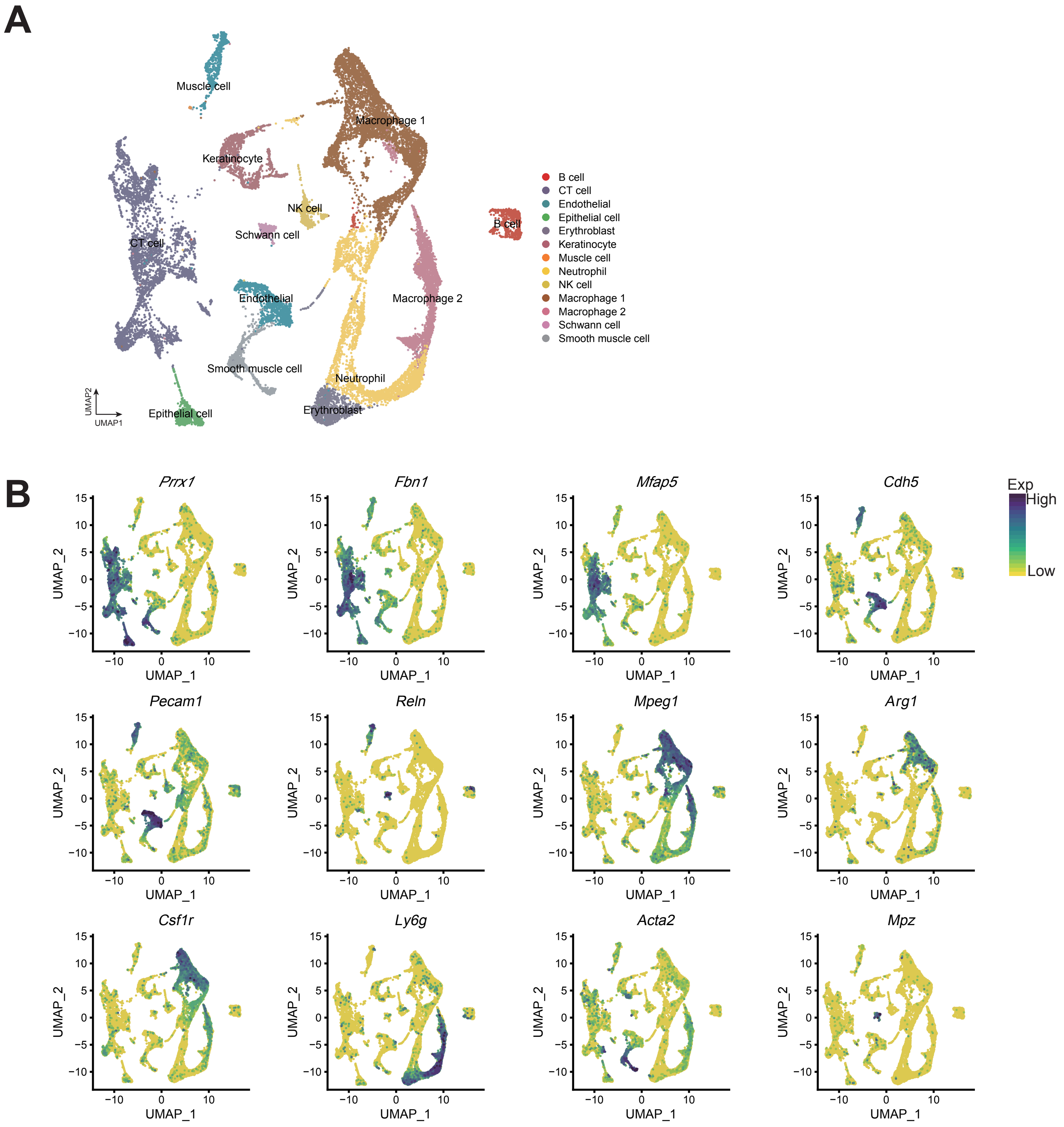
A
Muscle cell
Macrophage 1
Keratinocyte
NK cell
Schwann cell
CT cell
B cell
Endothelial
Macrophage 2
Smooth muscle cell
Neutrophil
Epithelial cell
Erythroblast
UMAP2
UMAP1
B cell
CT cell
Endothelial
Epithelial cell
Erythroblast
Keratinocyte
Muscle cell
Neutrophil
NK cell
Macrophage 1
Macrophage 2
Schwann cell
Smooth muscle cell
B
Prrx1
Fbn1
Mfap5
Cdh5
Pecam1
Reln
Mpeg1
Arg1
Csf1r
Ly6g
Acta2
Mpz
Exp
High
Low
UMAP_1
UMAP_2

Figure S5

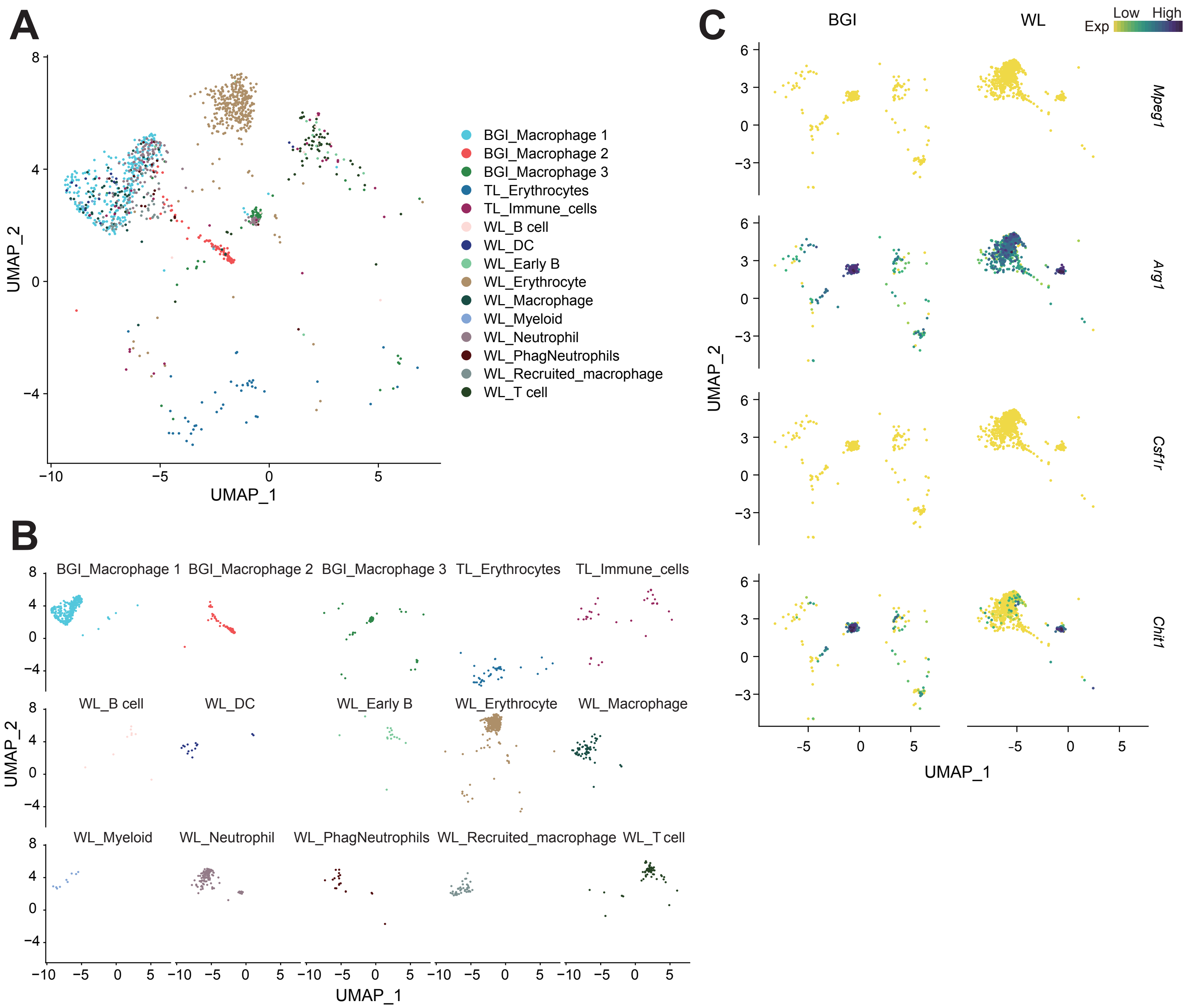

A
UMAP_2
UMAP_1
BGI_Macrophage 1
BGI_Macrophage 2
BGI_Macrophage 3
TL_Erythrocytes
TL_Immune_cells
WL_B cell
WL_DC
WL_Early B
WL_Erythrocyte
WL_Macrophage
WL_Myeloid
WL_Neutrophil
WL_PhagNeutrophils
WL_Recruited_macrophage
WL_T cell
B
C
BGI
WL
Exp
Low
High
Mpeg1
Arg1
Csf1r
Chit1

Figure S6

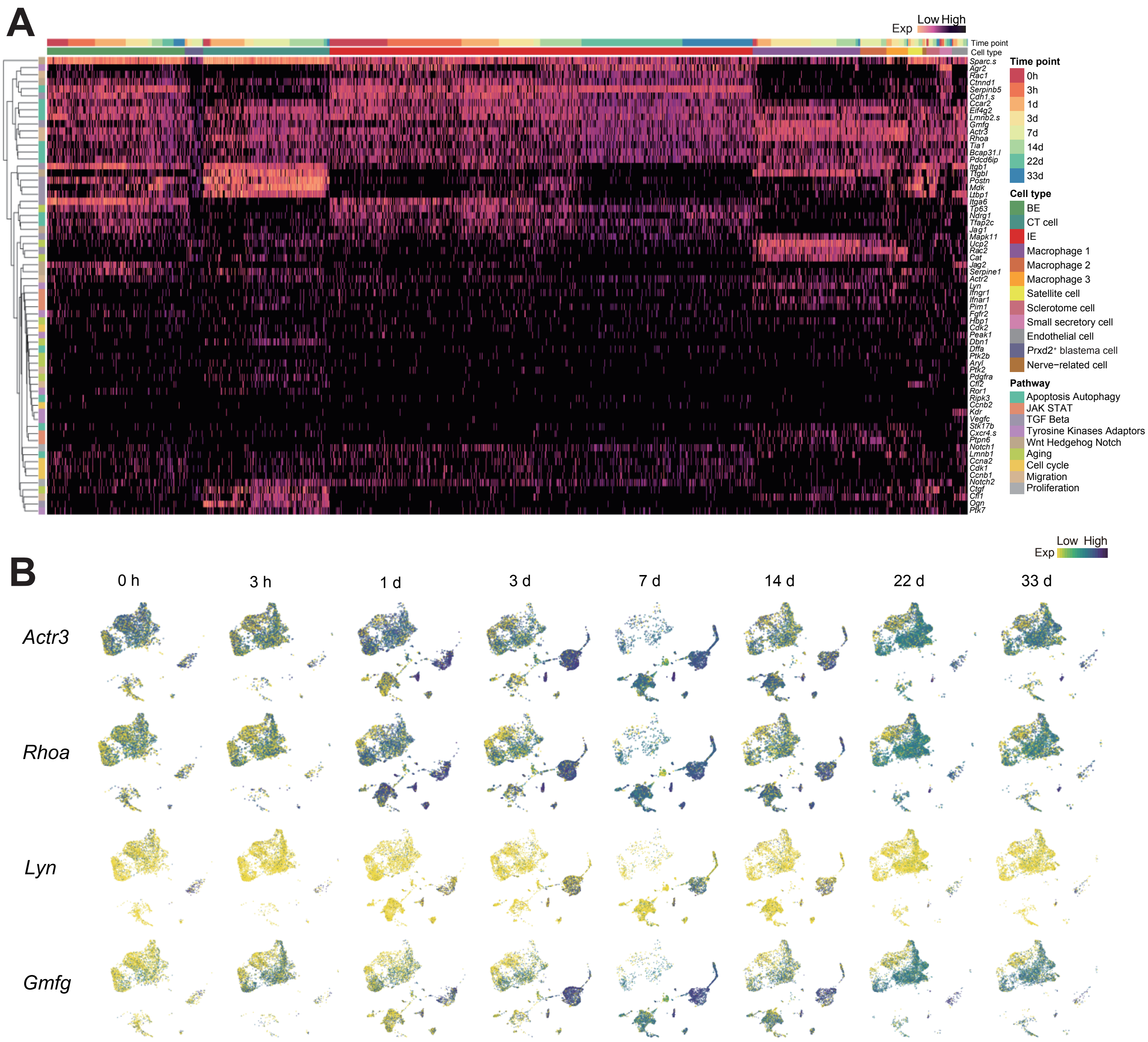
A
Exp
Low High
Time point
Cell type
Sparc.s
Agr2
Rac1
Ctnnd1
Serpinb5
Cdh1.s
Ccar2
Eif4g2
Lmnb2.s
Gmfg
Actr3
Rhoa
Tia1
Bcap31.l
Pdcd6ip
Itgb1
Tfgbi
Postn
Mdk
Ltbp1
Itga6
Tp63
Ndrg1
Tfap2c
Jag1
Mapk11
Ucp2
Rac2
Cat
Jag2
Serpine1
Actr2
Lyn
Ifngr1
Ifnar1
Pim1
Fgfr2
Hbp1
Cdk2
Peak1
Dbn1
Dffa
Ptk2b
Aryl
Ptk2
Pdgfra
Cfl2
Ror1
Ripk3
Ccnb2
Kdr
Vegfc
Stk17b
Cxcr4.s
Ptpn6
Notch1
Lmnb1
Ccna2
Cdk1
Ccnb1
Notch2
Ctgf
Cfl1
Ogn
Ptk7
Time point
0h
3h
1d
3d
7d
14d
22d
33d
Cell type
BE
CT cell
IE
Macrophage 1
Macrophage 2
Macrophage 3
Satellite cell
Sclerotome cell
Small secretory cell
Endothelial cell
Prxd2⁺ blastema cell
Nerve−related cell
Pathway
Apoptosis Autophagy
JAK STAT
TGF Beta
Tyrosine Kinases Adaptors
Wnt Hedgehog Notch
Aging
Cell cycle
Migration
Proliferation
B
Low High
Exp
0 h
3 h
1 d
3 d
7 d
14 d
22 d
33 d
Actr3
Rhoa
Lyn
Gmfg

Table S1. Quality Control Table of our scRNA-seq Data. Parameter names are listed in the first column, sampling stages are listed in the first row.

Table S2. Cell Type Identification Marker Genes of 41,376 Cells at 8 Stages During Adult Axolotl Limb Regeneration. Cell types are listed in the first column, marker genes for respective cell type identification are listed in the second column.

Table S3. Differentially Expressed Genes (DEG) Dataset of *Prdx2*$^{+}$ blastema Cells During Adult Axolotl Limb Regeneration. The first column is DEGs cluster number. The second column provides gene names or codes. The third to eighth columns provide average fold change (avg_logFC) and adjust *P* value (p_val_adj) data of listed genes between cells at different stages (1 d, 3 d, 7 d, 14 d). Stages with less than 50 *Prdx2*$^{+}$ blastema cells were removed. The first stage with more than 50 *Prdx2*$^{+}$ blastema cells was used as baseline stage for comparison. Data is saved as csv file.

Table S4. Differentially Expressed Genes (DEG) Dataset of Macrophage 3 During Adult Axolotl Limb Regeneration. The first column is DEGs cluster number. The second column provides gene names or codes. The third to eighth columns provide average fold change (avg_logFC) and adjust *P* value (p_val_adj) data of listed genes between cells at different stages (1 d, 3 d, 7 d, 14 d). Stages with less than 50 Macrophage 3 cells were removed. The first stage with more than 50 Macrophage 3 cells was used as baseline stage for comparison. Data is saved as csv file.
